# Catheter-Related Bloodstream Infection with *Rhizobium radiobacter* and *Pseudomonas oryzihabitans* Co-Infection: A Case Report and Literature Review

**DOI:** 10.3390/antibiotics15010028

**Published:** 2025-12-31

**Authors:** Hsien-Po Huang, Po-Yu Liu, Po-Hsiu Huang

**Affiliations:** 1 Division of Infectious Diseases, Department of Internal Medicine, Taichung Veterans General Hospital, Taichung 407, Taiwan; hsienpohuang@gmail.com (H.-P.H.); liupoyu@gmail.com (P.-Y.L.); 2 School of Medicine, National Yang Ming Chiao Tung University, Taipei 112, Taiwan; 3 Department of Post-Baccalaureate Medicine, College of Medicine, National Chung Hsing University, Taichung 402, Taiwan

**Keywords:** *Rhizobium radiobacter*, *Pseudomonas oryzihabitans*, catheter-related bloodstream infection, co-infection

## Abstract

**Background:** Catheter-related bloodstream infections (CRBSIs) caused by environmental organisms are uncommon, and polymicrobial cases are even rarer. **Methods:** We describe the first case of catheter-related bloodstream infection caused by two infrequent environmental organisms—*Rhizobium radiobacter* and *Pseudomonas oryzihabitans*—occurring as a co-infection. **Results:** The patient’s occupation involved frequent exposure to moist, soil-contaminated environments. Although these bacteria are often considered contaminants, they are capable of causing invasive infections such as bacteremia, which can be life-threatening. **Conclusions:** This case underscores the emerging pathogenic potential of *R. radiobacter* and *P. oryzihabitans* co-infection, particularly in patients with underlying malignancies or end-stage renal disease who have indwelling vascular devices, and highlights the importance of considering occupational and environmental exposures in the differential diagnosis of unusual pathogens.

## 1. Introduction

The most common pathogens responsible for catheter-related bloodstream infections are skin-derived organisms such as *Staphylococcus epidermidis* and *Staphylococcus aureus*. In contrast, catheter-related bloodstream infections caused by environmental bacteria are relatively rare.

*Rhizobium radiobacter* is a soil-dwelling, plant-associated bacterium widely distributed in agricultural environments, where it is primarily recognized as a phytopathogen. Human infections are uncommon and are thought to arise from environmental exposure, particularly through contact with soil, water, or contaminated medical devices [1]. Similarly, *Pseudomonas oryzihabitans* is an environmental Gram-negative bacillus that has been isolated from soil, water, and moist surfaces, including hospital environments and medical equipment. Although often regarded as a low-virulence or contaminant organism, it has been implicated in opportunistic infections, particularly in immunocompromised hosts or patients with indwelling intravascular devices [2]. These environmental reservoirs provide a plausible source for catheter contamination and subsequent bloodstream infection.

To our knowledge, although peritonitis caused by co-infection with *Rhizobium radiobacter* and *Pseudomonas oryzihabitans* has been reported in a patient receiving long-term peritoneal dialysis [3], catheter-related bloodstream co-infection involving both organisms has not previously been described. Here, we report the first documented case of a catheter-related bloodstream infection caused by co-infection with *R. radiobacter* and *P. oryzihabitans*, along with a review of the literature.

## 2. Case Presentation

A 60-year-old man with esophageal squamous cell carcinoma and lung metastases had been receiving regular chemotherapy via a chemoport for four months. He presented to the emergency department in August 2025 with a three-day history of fever and chills. He is a farmer and there was no family or travel history of note. On physical examination, his blood pressure was 133/73 mmHg, temperature 38.9 °C, pulse rate 104 beats/min, and respiratory rate 20 breaths/min. Examination of the head, eyes, ears, nose, and throat was unremarkable. There was no clubbing, cyanosis, lymphadenopathy, or icterus. The lungs were clear, and no cardiac murmurs were heard. Bowel sounds were active, and there was no abdominal tenderness. Initial blood investigations were unremarkable except for leukocytosis (11,070/mm^3^) with neutrophil predominance (92%), normocytic normochromic anemia (hemoglobin: 9.6 g/dL), and an elevated C-reactive protein (CRP) level of 5.9 mg/dL.

On the fifth day of admission, blood cultures obtained from both peripheral blood and the chemoport grew both *Rhizobium radiobacter* and *Pseudomonas oryzihabitans*, identified by matrix-assisted laser desorption/ionization-time-of-flight mass spectrometry (MALDI-TOF MS; BioMérieux, Marcy-l’Étoile, France). Identification by the MALDI-TOF MS system was based on a score indicating the confidence level of the results for each sample, with a score ≥ 2.0 indicating reliable identification at the species level. Antimicrobial susceptibility testing was performed by VITEK^®^2 (BioMérieux) and interpreted according to the Clinical and Laboratory Standards Institute (CLSI) breakpoints. The *R. radiobacter* isolate was susceptible to trimethoprim–sulfamethoxazole, ceftriaxone, cefepime, piperacillin–tazobactam, gentamicin, ciprofloxacin, and imipenem, but resistant to ceftazidime and amikacin. The *P. oryzihabitans* isolate was susceptible to all tested antibiotics, including trimethoprim–sulfamethoxazole, ceftriaxone, ceftazidime, cefepime, piperacillin–tazobactam, amikacin, gentamicin, ciprofloxacin, and imipenem. The same organisms with similar antimicrobial susceptibility profiles were isolated from blood cultures obtained from both peripheral blood and the chemoport.

The diagnosis of catheter-related bloodstream infection was established based on accepted clinical and microbiological criteria, including the isolation of the same organisms with identical antimicrobial susceptibility profiles from paired peripheral blood and catheter-drawn cultures, in the absence of an alternative infectious focus. In this case, blood cultures obtained from the chemoport became positive two hours earlier than those obtained from peripheral blood, thereby fulfilling the differential time to positivity (DTP) criterion for CRBSI [4]. These findings support the catheter as the most likely source of bloodstream infection, and intravenous piperacillin–tazobactam (4.5 g every 6 h) was initiated. The chemoport was subsequently surgically removed. Follow-up blood cultures obtained 72 h after catheter removal and initiation of antimicrobial therapy showed no bacterial growth. After 7 days of intravenous therapy, he was discharged with oral ciprofloxacin (500 mg every 12 h) for an additional 7 days.

## 3. Discussion

*Rhizobium* species (formerly classified under the genus *Agrobacterium*) are Gram-negative bacilli that are primarily plant pathogens responsible for tumorigenic diseases. The genus comprises five recognized species: *Rhizobium radiobacter*, *Rhizobium rhizogenes*, *Rhizobium rubi*, *Rhizobium undicola*, and *Rhizobium vitis*. Among these, *R. radiobacter* has emerged as an opportunistic human pathogen [1]. It has been implicated in various infections, including catheter-related bloodstream infections, dialysis-associated peritonitis, urinary tract infections, and pneumonia [5,6]. Overall, the prognosis for *R. radiobacter* infections is generally favorable.

Seven adult cases of catheter-related bloodstream infections caused by *R. radiobacter* were reported previously (Table 1). Exposure to soil was documented in only one of the seven cases. Removal of the catheter was regarded as the definitive treatment for this organism, as persistent or relapsing infections may occur if these sources are not eliminated. Five of the seven patients reported required catheter removal. Susceptibility of *R. radiobacter* to antibiotics varies, depending on the reporting center. Most cases are resistant to ceftazidime but respond well to appropriate antibiotic therapy. In our case, *R. radiobacter* was susceptible to all antibiotics tested except ceftazidime and amikacin.

*Pseudomonas oryzihabitans* (previously classified as *Flavimonas oryzihabitans*) is a Gram-negative bacillus. Although it has occasionally been isolated from the environment, the source of human infection has rarely been documented. Previous reported cases include bacteremia, central nervous system (CNS) infections, catheter-associated infections or device-related infections, sinusitis, wound infections, and skin infections, especially in immunocompromised patients in a hospital settings [2]. Only few cases of bacteremia have been reported due to this rare pathogen.

Five adult cases of catheter-related bloodstream infections caused by *P. oryzihabitans* were reported previously (Table 2). Exposure to soil was not documented in any of the five cases. Four of five patients reported required catheter removal. Susceptibility of *P. oryzihabitans* to antibiotics varies, depending on the reporting center. Most cases are resistant to cefazolin. In our case, *P. oryzihabitans* was sensitive to all antibiotics tested.

The review of the published literature suggests that most reported infections caused by *Rhizobium radiobacter* or *Pseudomonas oryzihabitans* have occurred in patients with underlying comorbidities, particularly malignancies or end-stage renal disease requiring long-term vascular access. Only four reported cases involved patients without these conditions, including two patients with acquired immunodeficiency syndrome, one patient with sickle-cell disease, and one with diabetes mellitus. The existing literature appears heterogeneous with respect to patient characteristics, clinical settings, and diagnostic methods, and potential underreporting cannot be excluded. Although the number of reported cases remains limited, these findings appear to indicate that infections due to these rare environmental organisms predominantly occur in hosts with significant underlying diseases or indwelling intravascular devices. The present case is in keeping with this observation, as the patient had an underlying malignancy and a long-term central venous catheter.

To date, only a single case of *R. radiobacter* and *P. oryzihabitans* co-infection has been reported, which involved peritonitis in a patient undergoing long-term peritoneal dialysis [1]. The present report is the first documented case of a catheter-related bloodstream co-infection caused by both pathogens. The patient’s occupational exposure to soil and humid environments may have facilitated colonization or contamination of the vascular access device, ultimately leading to bloodstream infection. Although direct environmental cultures were not performed, the clinical course and microbiological findings strongly support a catheter-related origin. The patient recovered completely following prompt catheter removal and targeted antibiotic therapy, underscoring the importance of both timely device removal and appropriate antimicrobial management in such infections.

## 4. Conclusions

We report the first case of catheter-related bloodstream infection caused by co-infection with *Rhizobium radiobacter* and *Pseudomonas oryzihabitans*. Although both organisms are environmental and infrequently encountered in clinical practice, previously reported cases—including the present one—suggest an association with patients who have significant underlying conditions, such as malignancies or end-stage renal disease, particularly in the presence of indwelling vascular devices. Early recognition, appropriate antimicrobial therapy, and timely catheter removal appear to be important components of successful management. In the absence of environmental sampling, the source could not be confirmed; however, this case underscores the importance of considering associated environmental bacteria in bloodstream infections among patients with occupational exposure to soil or water.

## Figures and Tables

**Table 1:** Clinical characteristics of adult patients with catheter-related bloodstream infections caused by *Rhizobium radiobacter*.

Year/ Country	Age/ Gender	Underlying Diseases	Soil Exposure History	Antibiotic Sensitivity	Antibiotic Resistance	Outcome	Catheter Removal	Reference
2003, USA	64, Male	Calf osteosarcoma	No	Cefepime, imipenem, ticarcillin/clavulanate, trimethoprim- sulfmethoxazole, minocycline, rifampin and all quinolones	Amikacin and tobramycin	Clinical resolution with ciprofloxacin and trimethoprim/sulfamethoxazole	Yes	[7]
2008, Taiwan	52, Male	Multiple myeloma	Yes	N/A	N/A	Clinical resolution with ceftriaxone	Yes	[5]
2009, Japan	75, Female	End stage renal disease	No	Ampicillin, cefotiam, cefmetazole, imipenem/cilastatin, meropenem, amikacin, minocycline and levofloxacin	Piperacillin, fosfomycin and ceftazidime	Clinical resolution with ampicillin/sulbactam	Yes	[8]
2010, India	51, Male	Diabetes Mellitus	No	Amikacin, cefepime, cefotaxime, ceftriaxone, ciprofloxacin, gatifloxacin, gentamicin, imipenem, levofloxacin, meropenem, piperacillin-tazobactum, tetracycline, ticarcillin-clavullanate, cotrimoxazole	Aztreonam	Clinical resolution with imipenem	No	[9]
2017, Italy	77, Male	End stage renal disease	N/A	Ceftazidime, levofloxacin	N/A	Clinical resolution with ceftazidime	No	[10]
2023, USA	70, Male	Acute myeloid leukemia	No	Amikacin, cefepime, gentamicin, levofloxacin, meropenem, piperacillin-tazobactam, tobramycin	Ceftazidime	Clinical resolution with cefepime	Yes	[11]
	47, Male	Chronic myeloid leukemia	No	Amikacin, cefepime, gentamicin, levofloxacin, meropenem, piperacillin-tazobactam, tobramycin	Ceftazidime	Clinical resolution with cefepime	Yes	
2025, Taiwan (This case)	60, Male	Esophageal cancer	Yes	Trimethoprim–sulfamethoxazole, ceftriaxone, cefepime, piperacillin–tazobactam, gentamicin, ciprofloxacin, and imipenem	Ceftazidime and amikacin	Clinical resolution with piperacillin–tazobactam	Yes	

Abbreviation: N/A, not available.

**Table 2:** Clinical characteristics of adult patients with catheter-related bloodstream infections caused by *Pseudomonas oryzihabitans*.

Year/ Country	Age/ Gender	Underlying Diseases	Soil Exposure History	Antibiotic Sensitivity	Antibiotic Resistance	Outcome	Catheter Removal	Reference
1991, USA	40, Female	Chronic myeloid leukemia	No	Ampicillin, sulfamethoxazole-trimethoprim, pipracillin, tobramycin, gentamicin, tetracycline, ceftazidime, ceftriaxone, and ciprofloxacin	Cefazolin	Clinical resolution with ceftriaxone	Yes	[12]
	37, Female	Sickle cell disease	No	Sulfamethoxazole-trimethoprim, gentamicin, tobramycin, ceftazidime, pipracillin, and ciprofloxacin	Cefazolin and cefoxitin	Clinical resolution with ceftriaxone	Yes	
	41, Male	Acquired immunodeficiency syndrome	No	Sulfamethoxazole-trimethoprim, gentamicin, tobramycin, ceftazidime, pipracillin, and ciprofloxacin	Cefazolin and cefoxitin	Clinical resolution with ceftazidime	Yes	
2000, Spain	30, Male	Acquired immunodeficiency syndrome	No	Broad-spectrum cephalosporins, aztreonam, imipenem, aminoglycosides, ciprofloxacin, and trimethoprim-sulfamethoxazole	Ampicillin, amoxicillin-clavulanic acid, and cefazolin	Clinical resolution with ciprofloxacin	Yes	[13]
2018, Italy	75, Male	End stage renal disease	No	Piperacillin/Tazobactam, Ceftazidime, Meropenem, Amikacin, Gentamicin, Ciprofloxacin, Levofloxacin	N/A	Clinical resolution with gentamicin plus meropenem	No	[14]
2025, Taiwan (This case)	60, Male	Esophageal cancer	Yes	Trimethoprim–sulfamethoxazole, ceftriaxone, ceftazidime, cefepime, piperacillin–tazobactam, amikacin, gentamicin, ciprofloxacin, and imipenem	N/A	Clinical resolution with piperacillin–tazobactam	Yes	

Abbreviation: N/A, not available.

## Data Availability

Data supporting the findings of this study are available from the corresponding author upon reasonable request.
